# Dual intersection syndrome of the forearm: a case report

**DOI:** 10.11604/pamj.2015.21.325.4105

**Published:** 2015-08-31

**Authors:** Bouchra Zhari, Meryem Edderai, Hassan Boumdine, Touriya Amil, Hassan En-nouali

**Affiliations:** 1 Imaging Department, Mohamed V Military Hospital, Rabat, Morocco

**Keywords:** Intersection syndrome, wrist pain, inflammatory tendinopathy of the wrist, peritenosynovitis

## Abstract

The intersection syndrome, described since the 19^th^ century, is an uncommon disorder associated with rubbing at the crossing point between the first dorsal compartment muscles and the radial wrist extensor muscles. Imaging modalities used to diagnosis this syndrome includes ultrasonography and magnetic resonance imaging. We reported a case of a 60-year-old man presented to our formation with painful swelling on the dorsum of the wrist and forearm. An MRI and an ultrasound were performed, and objectified a dual cross syndrome of the forearm.

## Introduction

Multiple conditions can cause wrist and forearm pain; the most common are de Quervain tenosynovitis and thumb carp metacarpal arthritis. Intersection syndrome (tenosynovitis of the radial wrist extensors) is common cause of wrist pain but under diagnosed, because of an atypical presentation of symptoms or because of a rarely unrecognized entity. It is a non-infectious inflammatory pathology located at the crossing point between the first and the second dorsal compartment muscles and the radial wrist extensor muscles. Its incidence is reported to be between 0,2% and 0,37% [1]. Patients with intersection syndrome complain of radial wrist or forearm pain. Symptoms may be exacerbated by repetitive wrist flexion and extension. The diagnosis is often made clinically but may also be found thanks to sonography and MRI.

## Patient and observation

**Figure 1:**
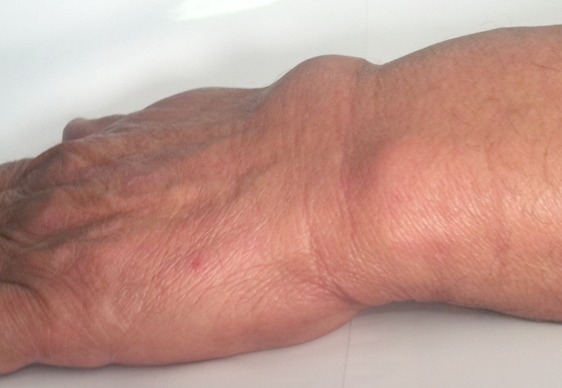
Showing swelling on the dorsal forearm

**Figure 2:**
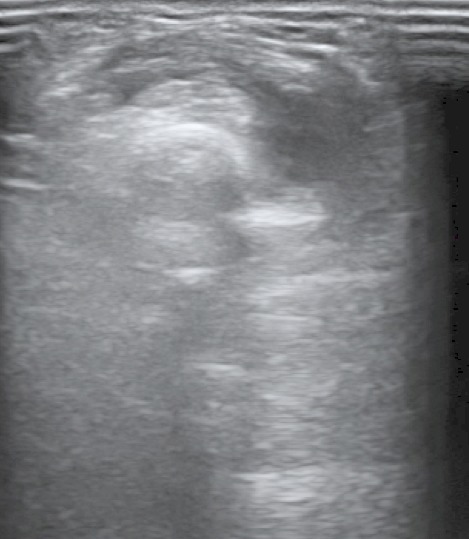
Ultrasonography wrist: axial section showing a peritendinous edematous thickening at the intersection of radial extensor tendons (ECRB, ECRL) with extensor pollicis longus (EPL)

**Figure 3:**
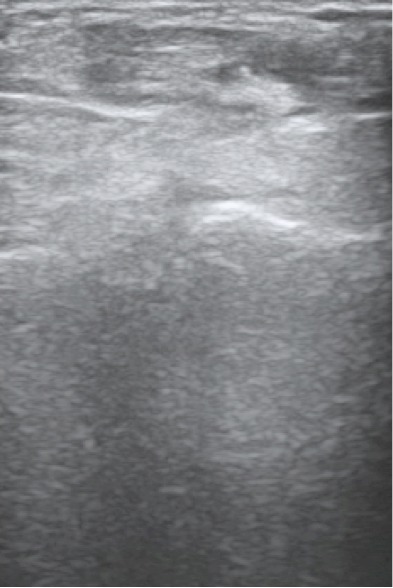
Ultrasonography wrist showing: tenosynovitis at the intersection of radial extensor tendons (ECRB, ECRL) with the abductor pollicis longus (APL) and extensor pollicis brevis (EPB)

**Figure 4:**
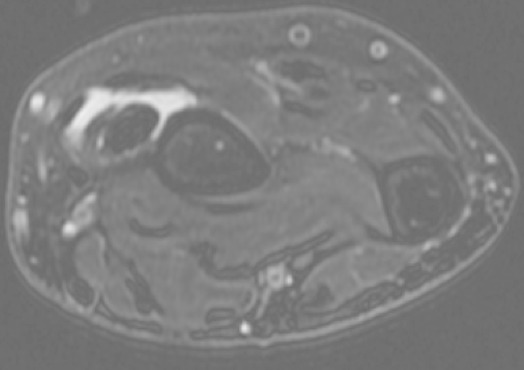
MRI axial view showing peritendinous effusion at the intersection of extensor carpi radialis brevis (ECRB), and extensor carpi radialis longus (ECRL) with extensor pollicis longus (EPL)

**Figure 5:**
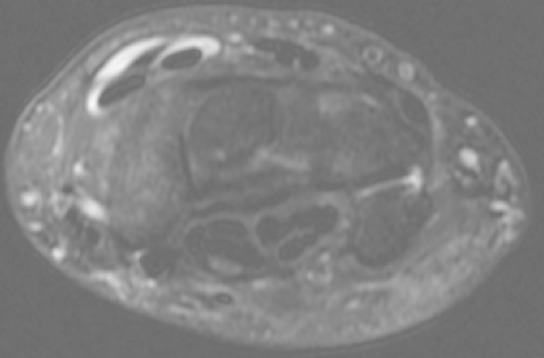
MRI axial view showing thickening and effusion peritendinous at the junction of ECRB and ECRL with extensor pollicis brevis (EPB) and abductor pollicis longus (APL)

**Figure 6:**
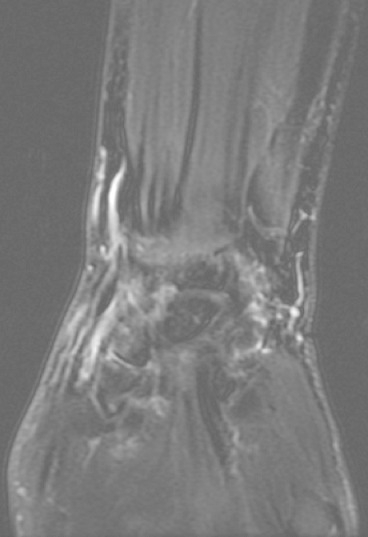
MRI coronal view showing view showing the extent of tenosynovitis

A 60-year-old man was admitted to our hospital suffering from pain and swelling on the dorsal forearm, 3 cm from the wrist (Figure 1). He sometimes feels a local crepitation while moving his thumb. The ultrasonographic and MRI exploration of the radial forearm (Figure 2, Figure 3, Figure 4, Figure 5, Figure 6) demonstrated the presence of a fluid collection around the sheaths of the following tendons: the extensor pollicis brevis (EPB), the abductor pollicis longus (APL) and the extensor pollicis longus (EPL), especially at the level of their intersection with the extensor carpi radialis brevis (ECRB) and the extensor carpi radialis longus (ECRL). A double cross of ECRL and ECRB tendons, with EPL at the bottom and EPB and APL at the upper side, was also demonstrated with an increase of effusion at these levels.

## Discussion

The intersection syndrome was described for the first time in 1841 by Velpeau, and it is also referred as adventitial bursitis, subcutaneous perimyositis, Abductor pollicislongus syndrome, peritendinitiscrepitans, cross-over syndrome, and oarsmen's wrist [2]. Yet most authors, believing that these names misstate the pathologic abnormality, prefer intersection syndrome. This term makes a clear statement about the location of the physical findings without being mislead about the pathologic anatomy [3].

It is common cause of wrist pain but under diagnosed, because of an atypical presentation of symptoms or because of a rarely unrecognized entity. It is a non-infectious inflammatory pathology located at the crossing point between the first and the second dorsal compartment muscles and the radial wrist extensor muscles [1]. There is no consensus about the pathophysiology of this condition, but this syndrome usually develops in patients involved in activities, such as rowing, canoeing, playing racket sports, horseback riding, and skiing, or occupations that require repetitive forceful flexion and extension of the wrist [3]. The prevalence of intersection syndrome was found to be 0.37%, in a study looking at 8,000 patients who presented with arm or hand pain in Thailand. In the general population, the prevalence varies between 0.20% and 0.37%. Another study by Palmer, looking at wrist injuries related to helicopter skiing in the Bugaboo mountains of British Columbia, suggested an incidence of intersection syndrome of 21% in that population of skiers [1].

At first we should define the proximal intersection syndrome, and the distal intersection syndrome. The pathogenesis of the first syndrome is explained by the musculotendinousjunctions of the first dorsal compartment tendons (abductor pollicislongus and extensor pollicisbrevis tendons) which intersect the second compartment tendons (extensor carpi radialislongus and extensor carpi radialisbrevis tendons) with an angle of approximately 60° and which is approximately 4cm far from the Lister′s tubercle [3]. Concerning the distal intersection syndrome there is tenosynovitis particularly of the extensor pollicislongus (EPL) tendon, where it crosses the extensor carpi radialislongus (ECRL) and brevis (ECRB) tendons. It is distinct from proximal intersection syndrome which occurs more proximally in the forearm at the intersection of the first and second extensor compartments. The particularity of our case is that it has a dual cross syndrome, distal and proximal. Clinically, there is usually a history of overuse through repetitive wrist flexion and extension, or less commonly direct trauma. Symptoms are a pain and a swelling over the dorsal radial forearm about 4 cm far from the wrist [4]. Severe cases may also manifest redness and a leathery crepitus. With a fiddling clinical observation and diagnosis of the mechanism of injury of the wrist and the identification of specific landmarks to locate the pain, we may consider intersection syndrome in the differential diagnosis of wrist pain such as: inflammatory forms of tenosynovitis (particularly De Quervain's disease), synovial cysts, infections, sprains involving the ligaments of the wrist, muscle strains, soft-tissue tumors, and Wartenberg's syndrome (entrapment of the sensory branch of the radial nerve) [4]. MRI can improve the evaluation of forearm and wrist symptoms.

A subtle but important distinction must be made between intersection syndrome and the De Quervain'sstenosing tenosynovitis, a pain in the wrist that involves thickening of the extensor retinaculum of the first dorsal compartment with stenosis of the canal that contains the APL and the EPB [2]. While the pain associated with the De Quervain'sstenosing tenosynovitis appears over the radial styloid, the pain of the intersection syndrome is located in the second dorsal compartment, several centimeters proximal to the radial styloid. Pain, edema, and crepitus that are found 4 to 8 cm far from the radial styloid are considered pathogneumonic for intersection syndrome.

The diagnosis is often made clinically but may also be found thanks to sonography and MRI. MRI is well suited to show the findings of intersection syndrome. The most important finding is the presence of peritendinous edema concentrically surrounding the second and the first extensor compartments, centredaround the point of crossover, about 4cm far from the Lister tubercle. Sometimes the edema may extend as far as the radiocarpal joint [3]. It may happen that the intersection syndrome may have findings of an associated reactive tenosynovitis. Mild subcutaneous edema adjacent to the intersection point is also a feature, probably resulting from surrounding hyperemia. Findings of tendinosis (thickening and signal abnormality) may not be present, reflecting principally a peritendinous phenomenon [3].

The treatment is similar to the one given for overuse injuries. Conservative measures are the first line of treatment. Symptoms resolve within 2 to 3 weeks for 60% of patients with rest, administration of non-steroidanti-inflammatory drugs, and splinting. Surgery is indicated typically only for patients not responding to therapy. When conservative treatment fails, a tenosynovectomy and a fasciotomy of abductor pollicislongus can be performed.

## Conclusion

Intersection syndrome is an overuse disorder of the dorsal distal forearm, presenting with particular symptoms and signs that may be clinically misdiagnosed. The diagnosis is made on the basis of clinical findings, which must be confirmed by imaging studies (US and MRI). It may be discovered only after an MRI especially during acute or sub acute periods. Thus, radiologists must keep in mind this possibility while diagnosing wrist pains because a clinical diagnosis could be difficult in such cases.

## References

[1] Gael Jean Yonnet MD (2013). Intersection Syndrome in a Handcyclist: case report and literature review. Top Spinal Cord Inj Rehabil..

[2] Montechiarello S, Miozzi F, D'Ambrosio I, Giovagnorio F (2010). The intersection syndrome: ultrasound findings and their diagnostic value. J Ultrasound..

[3] Costa CR, Morrison WB, Carrino JA (2003). MRI features of intersection syndrome of the forearm. AJR Am J Roentgenol..

[4] Lee RP, Hatem SF, Recht MP (2009). Extended MRI findings of intersection syndrome. Skeletal Radiol..

